# Polyvinylpyrrolidone-Capped Silver Nanoparticle Inhibits Infection of Carbapenem-Resistant Strain of *Acinetobacter baumannii* in the Human Pulmonary Epithelial Cell

**DOI:** 10.3389/fimmu.2017.00973

**Published:** 2017-08-16

**Authors:** Vishvanath Tiwari, Monalisa Tiwari, Vandana Solanki

**Affiliations:** ^1^Department of Biochemistry, Central University of Rajasthan, Ajmer, India

**Keywords:** *Acinetobacter baumannii*, polyvinylpyrrolidone-capped silver nanomaterial, host–pathogen interaction, carbapenem resistance, silver nanomaterial

## Abstract

*Acinetobacter baumannii*, an opportunistic ESKAPE pathogen, causes respiratory and urinary tract infections. Its prevalence increases gradually in the clinical setup. Pathogenicity of *Acinetobacter* is significantly influenced by its ability to infect and survive in human pulmonary cells. Therefore, it is important to study the infection of *A. baumannii* in human pulmonary host cell (A-549), monitoring surface interacting and internalized bacteria. It was found that during infection of *A. baumannii*, about 40% bacteria adhered to A-549, whereas 20% got internalized inside pulmonary cell and induces threefold increase in the reactive oxygen species production. We have synthesized polyvinylpyrrolidone (PVP)-capped AgNPs using chemical methods and tested its efficacy against carbapenem-resistant strain of *A. baumannii*. PVP-capped silver nanoparticles (PVP-AgNPs) (30 µM) have shown antibacterial activity against carbapenem-resistant strain of *A. baumannii* and this concentration does not have any cytotoxic effect on the human pulmonary cell line (IC_50_ is 130 µM). Similarly, PVP-AgNPs treatment decreases 80% viability of intracellular bacteria, decreases adherence of *A. baumannii* to A-549 (40 to 2.2%), and decreases intracellular concentration (20 to 1.3%) of *A. baumannii*. This concludes that PVP-AgNPs can be developed as a substitute for carbapenem to control the infection caused by carbapenem-resistant *A. baumannii*.

## Introduction

*Acinetobacter baumannii*, an ESKAPE pathogen, causes pneumonia, urinary tract infections, and respiratory infections, and its prevalence in clinical setup increases with time (1, 2). ESKAPE pathogen causes hospital-acquired infection and includes *Enterococcus faecalis, Staphylococcus aureus, Klebsiella pneumoniae, A. baumannii, Pseudomonas aeruginosa*, and *Enterobacter* species. The lethality of *A. baumannii* is due to the development of resistance against most of the antibiotics used to treat it. Resistance against carbapenem (2–6), the most effective β-lactams antibiotic, used against *Acinetobacter*, is one of the major concerns. Inflammation in lung is one of the important symptoms of pneumonia caused by *A. baumannii* resulting in epithelial barrier destruction (7). Interaction between *A. baumannii* and human pulmonary cells (alveolar epithelial) leads to infection because of its adherence and invasion into these cells (8–10) and induces cellular death (8). Pathogenicity of *Acinetobacter* is significantly influenced by its ability to survive in the human pulmonary cells. Therefore, it is important to study the interaction of *A. baumannii* with human pulmonary host cell.

AgNPs (silver nanoparticles) have antimicrobial property against diverse microbes, probably due to their different mechanisms of antimicrobial action (11, 12). Polyvinylpyrrolidone (PVP) is a neutral stabilizer and less sensitive to surface charge screening processes like pH change and ionic strength (13). Recent reports have shown that PVP-capped AgNPs are more stable than other AgNPs (14, 15). PVP-capped AgNPs are also less toxic to the mammalian cells (16). Likewise, interaction of PVP-capped silver nanoparticles (PVP-AgNPs) with serum proteins affects its *in vivo* antimicrobial activity. It has been reported that PVP-capped AgNPs have better *in vivo* antimicrobial activity than other capped AgNPs (17). Hence, they can be used as a substitute to the carbapenem. Therefore, in the present study, we have monitored the infection of carbapenem-resistant strain of *A. baumannii* to the human pulmonary (alveolar epithelial) cell line A-549. Further, we have also monitored the surface interacting and internalized bacteria and tested the efficacy of PVP-capped AgNPs on the infection of *A. baumannii* in A-549 cell line. The present result might help to understand the infection caused by *A. baumannii* to human pulmonary cell as well as control of infection caused by it using the PVP-capped silver nanomaterial.

## Materials and Methods

### Bacterial Strain

All the work related to cell line and bacteria were performed aseptically, under laminar airflow. Clinical strain RS-307 of *A. baumannii* was grown in Luria-Bertani broth (LB) media at 37°C till it reached optical density to 0.6 OD. After sufficient bacterial growth, streaking was done on the Luria agar plate to isolate the single bacterial colony. Single colony was used for suspension culture. Prior to coinfection, bacterial culture was centrifuged and re-suspended in PBS to remove deleterious effects of toxins (cytotoxins) present in the culture.

### Human Pulmonary Cell Line Culture

The human alveolar basal epithelial cell line A-549 (purchased from Cell repository, NCCS, Pune) was cultured in high glucose Dulbecco’s Modified Eagle Medium (DMEM) supplemented with 10% heat inactivated fetal bovine serum albumin (heat inactivation was performed at 56°C for 30 min), amphotericin B (2.5 µg/ml), vancomycin (50 µg/ml), gentamicin (50 µg/ml), and 1% HEPES in a humidified, 5% CO_2_ at 37°C. Passage of A-549 human pulmonary cell line in 25 cm^2^ flask was done after every 2–3 days intervals when they were confluent. The cells were seeded for 24 h in 96 well plates for MTT and LDH assay and 6-well plates for coinfection with the *A. baumannii*.

### Preparation of Cell Line and Bacterial Culture for Infection

Confluent A-549 cells flask was washed twice with PBS buffer (pre-warm at 37°C). The cells were detached with the help of 0.05% trypsin-EDTA and complete media was added. The suspension was centrifuged and cells pellet were re-suspended in the fresh DMEM medium containing 10% fetal bovine serum without antibiotics, at a concentration of 2 × 10^5^ cells/ml. Simultaneously, bacterial culture was inoculated in 100 ml of LB broth and was grown at 37°C with vigorous shaking (180 rpm) till optical density reached to 0.6. We have counted colony-forming unit (CFU) of the bacteria before coinfection.

### Infection of A-549 Cells by RS-307 Strain of *A. baumannii*

A-549 cells were grown at 37°C in 5%CO_2_ till they reached at least 90% confluency without contamination. The cells were washed with pre-warm PBS. 1 ml of fresh medium supplemented with 10% serum without antibiotics was added to each well. The control wells were prepared by adding fresh medium without A-549 cells in three wells. These wells were used as a blank for addition of bacterial strains. Coinfection was achieved by adding an aliquot of each bacterial culture to each well containing A-549 cells (in triplicate) and in the blank. This coinfection represents MOI (multiplicity of infection) of 3:1 (bacteria, 1.2 × 10^7^: cells, 0.4 × 10^7^). Infected A-549 cells were incubated in incubator for 24 h at 37°C with 5% CO_2_.

### CFU Counting of Different Stages of the Infections

We have counted bacterial populations of four different stages of infection, i.e., before coinfection, remain non-interact during coinfection (suspension culture in DMEM media which contain the free bacteria), interacting or adhered bacteria on the surface of A-549 (which were isolated after mixing of cell pellet in the PBS and centrifugation, as explained in next section) and internalized bacteria into A-549 (which were isolated after homogenization). The CFU was determined for above four stages.

### Protein Extraction

After 24 h incubation, washing was done 3 times with pre-warm PBS. 1 ml of PBS (containing 0.1 mM protease inhibitor PMSF) was added. Adhered bacteria were detached and centrifuged at 3000g for 5 min to separate the loosely adhered bacteria. The pellet was dissolved in 1 ml PBS (with 0.1 mM PMSF). The dissolved pellet was homogenized for 1 min. Homogenized sample were centrifuged at 7000g for 10 min. The supernatant was stored at −80°C containing A-549 cell lines proteins. The pellet (containing internalized bacteria) was suspended in 1 ml PBS with 0.1 mM PMSF. This suspended pellet was sonicated for three cycles of 30 s (20 kHz, 130 W) with the interval of 1 min. The sonicated samples were centrifuged at 10000g for 10 min. The supernatant contained bacterial protein and was stored at −80°C. The samples were collected before homogenization and sonication, and used for bacterial CFU counting that represent adhered and internalized bacteria, respectively. Protein concentrations were measured for bacterial samples (internalized and adhere) and cell line (before and after bacterial infection) using Bradford methods.

### Preparation of PVP-Capped AgNPs and *In Vitro* Antimicrobial Activity Test

Polyvinylpyrrolidone-capped AgNPs were prepared as per our published methods (12). The size and zeta-potential of synthesized PVP-capped AgNPs were monitored using dynamic light scattering-based particle analyzer. *In vitro* susceptibility of PVP-AgNPs (30 µM) was performed on carbapenem-resistant strain of *A. baumannii* using disc diffusion assay and growth kinetics analysis as per our published protocol (18).

### Treatment of PVP-Capped AgNPs and CFU Counting for Different Stages of the Infections

For the treatment, four T-25 flasks were prepared. First flask was used as a control for cell line, second flask was used for bacterial infection, third flask was for AgNPs treatment after bacterial infection, and fourth flask as negative control for AgNPs treatment. All four flasks were incubated at 37°C for 24 h with 5% CO_2_. Coinfection was achieved by adding 1.2 × 10^7^ CFU that represents MOI of 3:1 (bacteria, 1.2 × 10^7^: cells, 0.4 × 10^7^). Infected A-549 cells were incubated in the CO_2_ incubator at 37°C for 24 h with 5% CO_2_. 500 µl of culture media was taken for counting non-interacting bacteria. The media was discarded gently and flasks were washed mildly two times by 2 ml PBS. All the washed cell lines were collected in 1 ml PBS. This suspended cell line mixture contains surface interacting bacteria and cell line (with or without engulf bacteria). This mixture was centrifuged at 3000g for 5 min and supernatant was used for CFU counting of interacting bacteria. The collected samples were homogenized for 3 min (1 min pulse each) and centrifuged at 7,000g for 10 min. The supernatant contains cell line proteins and pellet contains engulfed bacteria. The pellet was dissolved in 500 μl PBS and used for CFU counting of engulf bacteria. Suspended bacteria was further sonicated and centrifuged. The supernatant contained bacterial proteins. The cell line proteins and bacterial proteins of different conditions were used for the SDS-PAGE analysis.

### Cytotoxic Effect of PVP-Capped AgNPs on the A-549 Cell Line and Survival of *A. baumannii* after PVP-AgNPs Treatment

Cytotoxic effect of PVP-capped AgNPs (15–300 µM) on the A-549 cell line was investigated using MTT (EZcount™ MTT Cell Assay Kit) and LDH assay (EZcount™ Lactate Dehydrogenase Cell Assay Kit) to find the non-toxic dose as well as IC_50_ concentration of PVP-AgNPs.

### Cytotoxic Effect of PVP-AgNPs on the Internalized *A. baumannii*

Viability of *A. baumannii* in infected A-549 cell line was monitored using MTT assay. The triplicated experiment was performed on A-549 alone, A-549 cell line infected with *A. baumannii*, and *A. baumannii*-infected A-549 cell line treated with PVP-AgNPs. The viable internalized bacteria were calculated by subtracting total viability of infected A-549 cell line with *A. baumannii* by the viability of uninfected A-549 cell line.

### Reactive Oxygen Species (ROS) Formation during Infection and PVP-Capped AgNPs Treatment

Reactive oxygen species production was monitored by nitroblue tetrazolium (NBT) reduction assay for uninfected A-549 cell lines, *A. baumannii* infected A-549 cell lines (A-549), and *A. baumannii*-infected cell lines after PVP-AgNPs treatment. NBT stock solution (10 mg/ml) was prepared in distilled water and kept in dark at 4°C. A working NBT solution (0.3%) was prepared in culture medium just before utilization. It was diluted three times in each well, giving a final concentration of 0.1% NBT in the well. 50 µl supernatants were eliminated when the cells were adhered and replaced it by 50 µl of 0.3% NBT working solution. After 2 h of incubation, supernatant was removed and cells were fixed by adding of 200 µl of methanol and washed twice with 70% methanol, then dried. The formazan deposits were solubilized in 120 µl 2 M KOH and 140 µl DMSO. After vigorous mixing of all the contents of wells, absorbance was recorded at 620 nm using UV-Vis spectrophotometer. To remove the contribution coming from the ROS produced by *A. baumannii*, a bacterial control was also taken.

## Results

The adherence and persistence ability of *A. baumannii* on the host cell is central to its pathogenicity. To cause host cell infections, bacteria first colonize on the surface of the host. They express different types of adhesin molecules for attachment to host cells. These adhesins bind the surface soluble proteins of the host and act as a bridge between host and bacteria. Adherence is the first most important step than invasion and secretion of toxins. Adhered bacteria invade into the host cell and causes infection. In this study, we have used RS-307 strain of *A. baumannii*, which is a multidrug resistant strain of *A. baumannii* and have high MIC (>64 μg/ml) for imipenem (a carbapenem), and A-549 cell line, which is a human pulmonary (alveolar epithelial) cells. This cell line has been chosen as *A. baumannii* causes pneumonia that is associated with lungs.

### A-549 Cell Line Cell Death Induced by *A. baumannii* Infection

**Figure 1:**
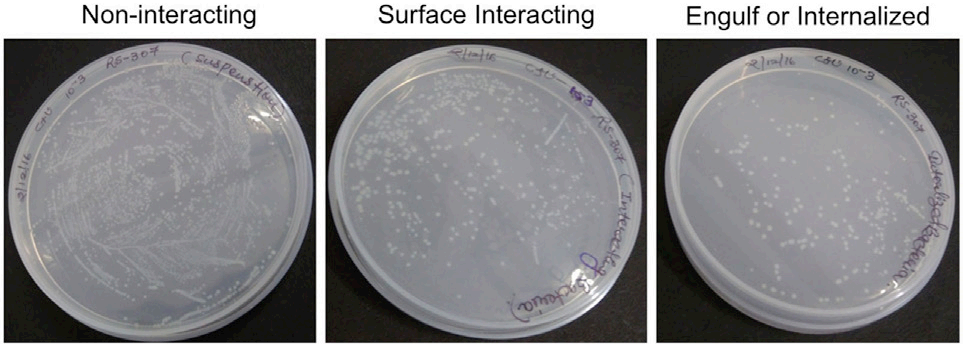
Counting of colony-forming unit of non-interacting, surface interacting, and engulf or internalized bacteria cultured on Luria-Bertani Agar at 10^−3^ dilution. The triplicate experiments were performed and result of one experiment is shown.

**Figure 2:**
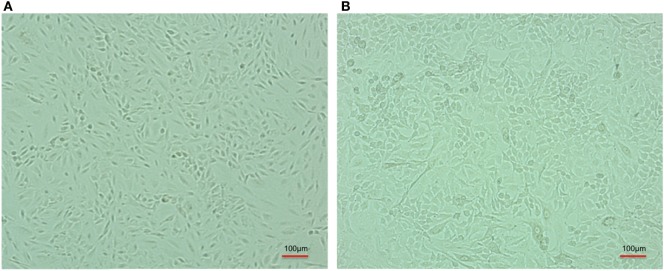
Comparative cell line image of A-549 cell line in the absence **(A)** and coinfected with carbapenem-resistant strain, RS 307 of *Acinetobacter baumannii* **(B)**. The experiments were performed in quadruplet. Infection leads to the lysis of A-549 cell.

After infection, A-549 cell lines were incubated for 24 h and numbers (CFU) of *A. baumannii* were monitored for interacting or adhered bacteria on the surface of A-549 and internalized bacteria into A-549. The CFU result is presented in the Table 1. Table shows that about 41% bacteria remain un-interacted, 39.6% bacteria adhered to the surface of A-549 cells, and 18.4% bacteria were engulfed or internalized. The CFU results of these stages are presented in the Figure 1. The infection of A-549 cell line with the *A. baumannii* leads to the lysis of the A-549 cell lines and also changes the morphology of A-549 cell lines that can be seen in Figure 2.

**Table 1:** Comparative display of colony-forming unit (CFU) counting at different stages of *A. baumannii* infection in absence and presnce of PVP-capped silver nanoparticles (PVP-AgNPs).

Condition for the culture	Bacteria used for coinfection	Non-interacting bacteria CFU	Surface interacting bacteria CFU	Engulf or interlized bacteria CFU
A-549 + bacteria	1.2 × 10^7^ (100%)	0.50 × 10^7^ (41.66%)	47.6 × 10^5^ (39.66%)	22.1 × 10^5^ (18.41%)
A-549 + bacteria + PVP-AgNPs	1.2 × 10^7^ (100%)	1.15 × 10^7^ (95.83%)	2.6 × 10^5^ (2.17%)	1.5 × 10^5^ (1.25%)

*The experiment was performed in triplciates and their mean values are shown*.

### *In Vitro* Susceptibility of Carbapenem-Resistant *A. baumannii* against PVP-Capped AgNPs

**Figure 3:**
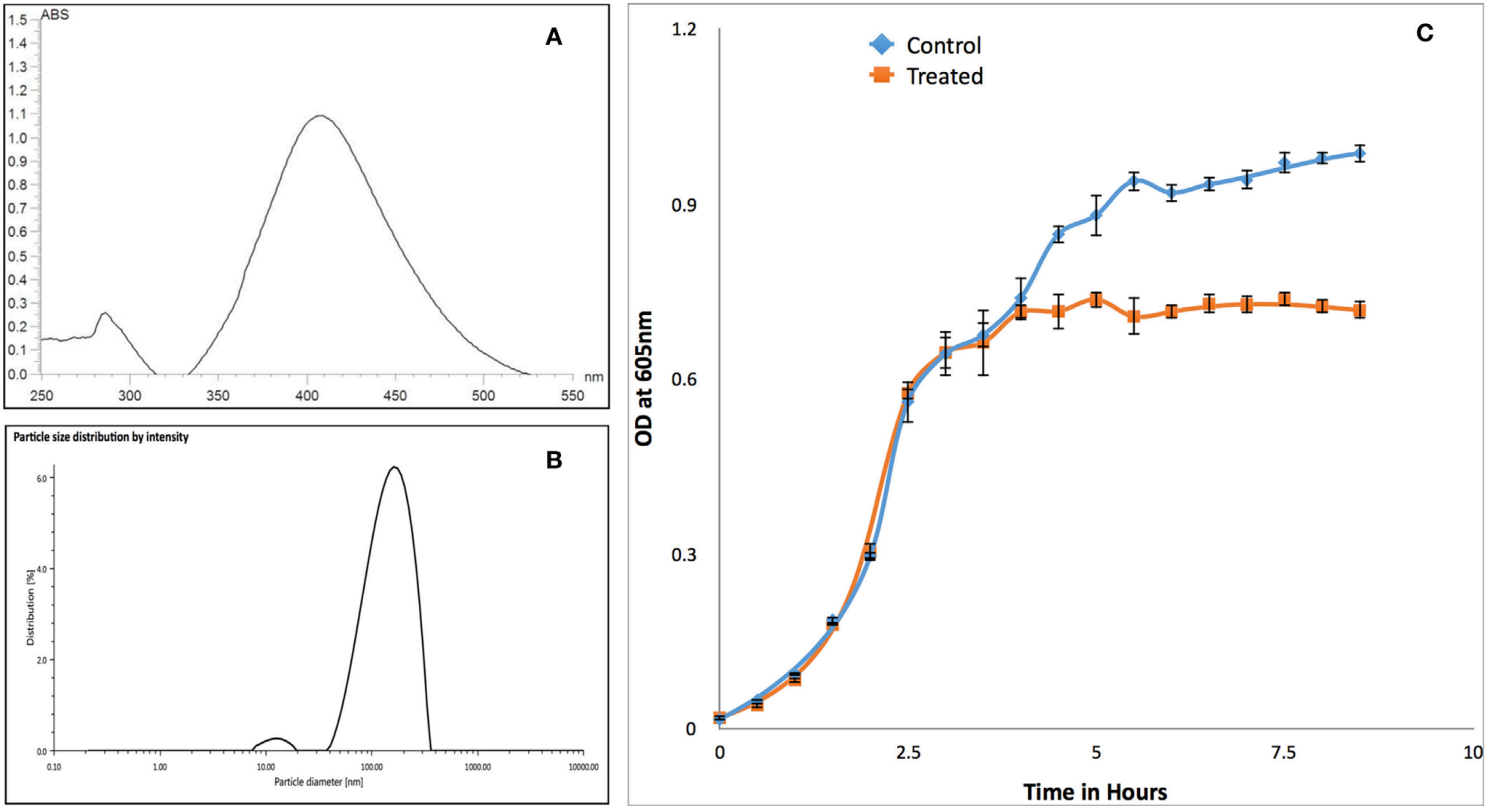
Characterization of PVP-capped AgNPs using UV-Vis spectroscopy **(A)** and dynamic light scattering **(B)**. Similarly section **(C)** showed comparative growth curve of RS-307 strain of *Acinetobacter baumannii* in the absence and presence of PVP-capped silver nanoparticles. Growth curve experiments were performed in triplicate and data are presented as mean ± SD.

Synthesized PVP-capped AgNPs have maximum absorbance peak around 400 nm for AgNPs (Figure 3A) and size of 133 nm with polydispersity index of 23% (Figure 3B). Similarly, zeta-potential of nanoparticle was found to be −34 mV. Growth kinetics of RS-307 strain of *A. baumannii* was determined in the absence and presence (30 µM) of PVP-capped AgNPs. Optical density was measured at 605 nm at an interval of 30 min using UV-Vis spectrophotometer. Growth curves were prepared using absorption data. The experiment was performed in triplets for untreated and treated samples and average value was used to prepare the plot. Relative growth curves were prepared for comparison purpose. The result showed that PVP-capped AgNPs showed good antimicrobial activity against carbapenem-resistant strain of *A. baumannii* (Figure 3C).

### Cytotoxicity Test of PVP-Capped AgNPs on A-549 Confirm Its Non-Toxic Nature at Its Inhibitory Concentration

**Figure 4:**
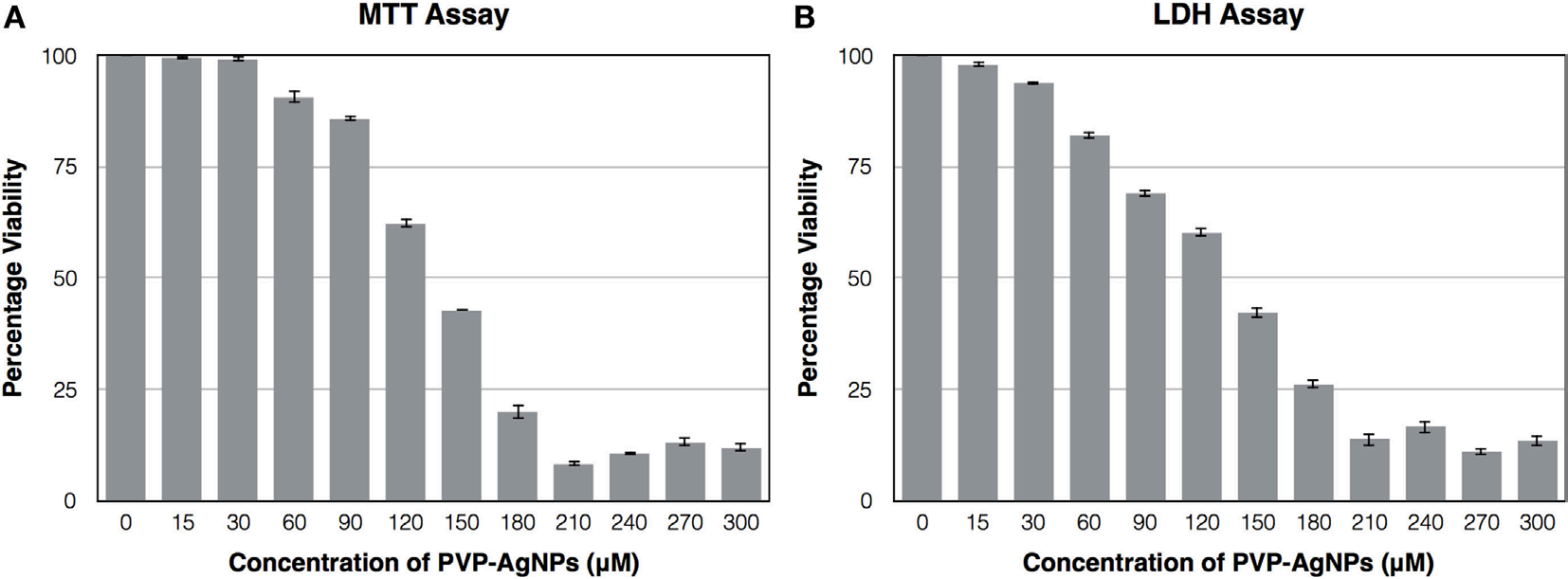
Effect of different concentration of PVP-capped AgNPs on the viability of A-549 cell line using MTT assay **(A)** and LDH assay **(B)**. The experiments were performed in triplicate and data are presented as mean ± SD.

The cytotoxic effect of PVP-capped AgNPs on the human pulmonary epithelial cell lines (A-549) was identified using MTT Assay and LDH assay. Figure 4 showed that the concentration at which it showed bactericidal activity has no cytotoxicity. The IC_50_ value for PVP-AgNPs against A-549 cell line was found to be 130 µM, which is four times higher than the concentration showing antimicrobial activity. The 30 µM PVP-AgNPs also does not have hemolytic activity.

### PVP-Capped AgNPs Prevent A-549 Cell Line Cell Death Induced by *A. baumannii*

**Figure 5:**
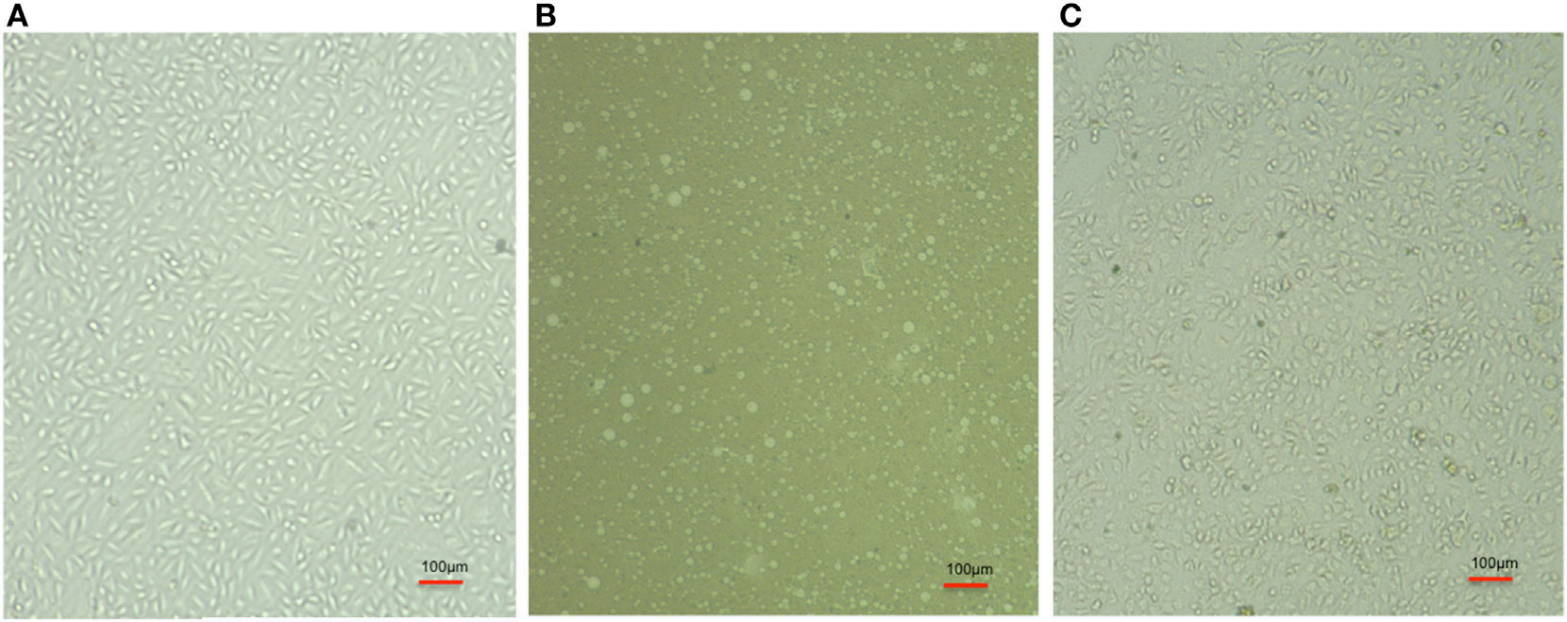
Comparative cell line image of A-549 cell line **(A)**, *Acinetobacter baumannii* infected A-549 cell line **(B)**, and PVP-capped silver nanoparticles-treated *A. baumannii* infected A-549 cell line **(C)**. Bulbs seen in panel **(B)** represent lysed A549 cell after *A. baumannii* infection. The triplicate experiments were performed and result of one experiment is shown.

The infected cell line of A-549 was treated with PVP-AgNPs. The infection leads to the death of A-549 cell line (Figure 5B) but PVP-AgNPs protects pulmonary cell line (A-549) from the infection of *A. baumannii* (Figure 5C). The morphologies of PVP-capped AgNPs-treated A-549 cell line (Figure 5C) are very similar to the uninfected cell lines (Figure 5A). Therefore, it can be suggested that presence of PVP-AgNPs prevent infection of carbapenem-resistant strain of *A. baumannii* in A549 cell line.

### PVP-Capped AgNPs Decreases Attachment and Internalization of *A. baumannii* into A-549

Colony-forming unit counting in the absence and presence of PVP-AgNPs showed that PVP-AgNPs reduce the number of surface attached bacteria from about 40 to 2.2%. The number of intracellular bacteria was also reduced from 18 to the 1.25%. Therefore, we can say that PVP-AgNPs reduces the attachment and internalization of *A. baumannii* on the pulmonary cell model.

### PVP-Capped AgNPs Decrease the Viability of Intracellular *A. baumannii*

Intracellular viability was calculated by subtracting absorbance of infected A-549 cell line with absorbance of uninfected A-549 cell lines. The result of viability test showed that there is 80% decrease in the viability of intracellular *A. baumannii* after treatment (OD 0.12) with 30 µM PVP-AgNPs as compared to untreated sample (OD 0.56). It also enlightened that PVP-AgNPs not only inhibit free *A. baumannii* but also inhibit the intracellular *A. baumannii*.

### ROS Production by A549 Cell Line after *A. baumannii* Infection

**Figure 6:**
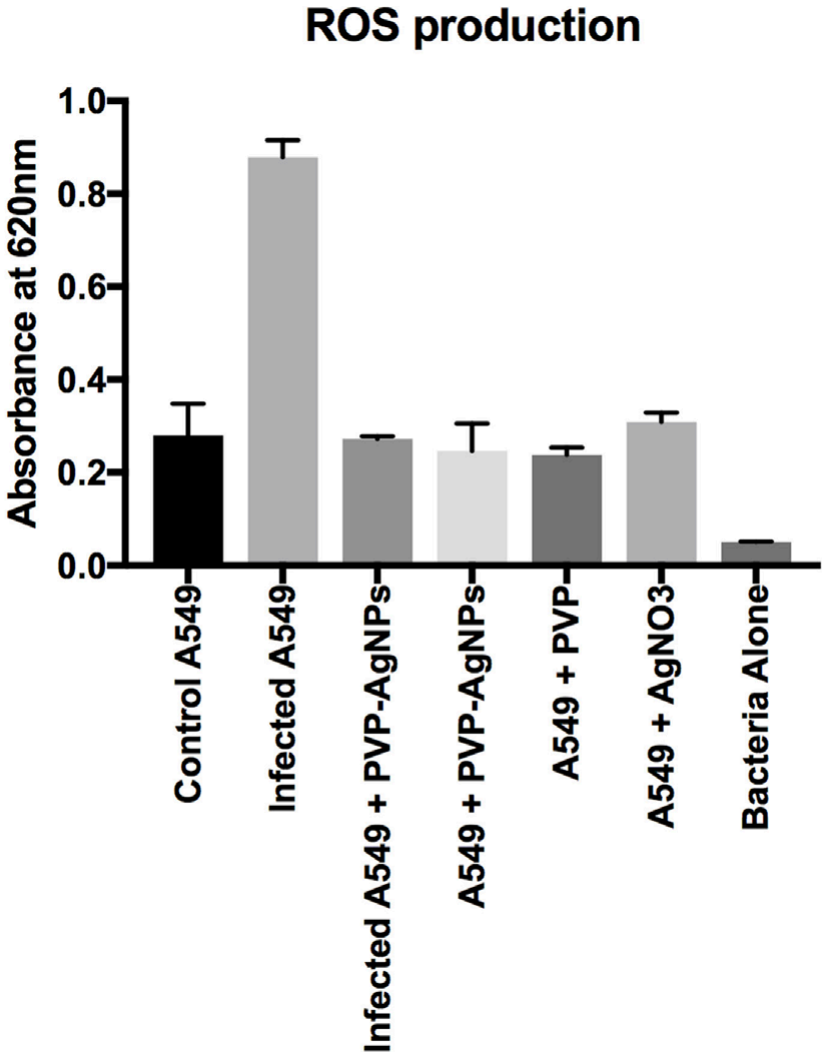
Reactive oxygen species (ROS) production by A-549 under different conditions of infection and treatment. ROS production by A-549 under infected condition was calculated by subtracting ROS produced by bacteria alone. Experiment was performed in triplicate and data are presented as mean ± SD.

Reactive oxygen species are used by pulmonary epithelial cells against pathogens (19). ROS can kill pathogens (such as *A. baumannii)* directly by causing oxidative damage to biomolecules or indirectly by stimulating pathogen elimination by various non-oxidative mechanisms (19). Therefore, we have monitored the ROS production during the infection as well as treatment. Result (Figure 6) showed that *A. baumannii* infection promotes threefold increase (OD at 620 nm is 0.83 for infected and 0.27 for uninfected) in the production of ROS. This is correlated with the previous result, which showed that ROS production increases in pulmonary epithelial cells after bacterial infection. The treatment of PVP-AgNPs shifted the ROS level to normal, which also confirms that approximately all the intracellular bacteria were killed by PVP-AgNPs. The result is correlated with the bacterial viability assay, which also confirms that about 80% intracellular bacteria were killed by the PVP-AgNPs.

### PVP-Capped AgNPs Treatment Changes the SDS Protein Profiling

**Figure 7:**
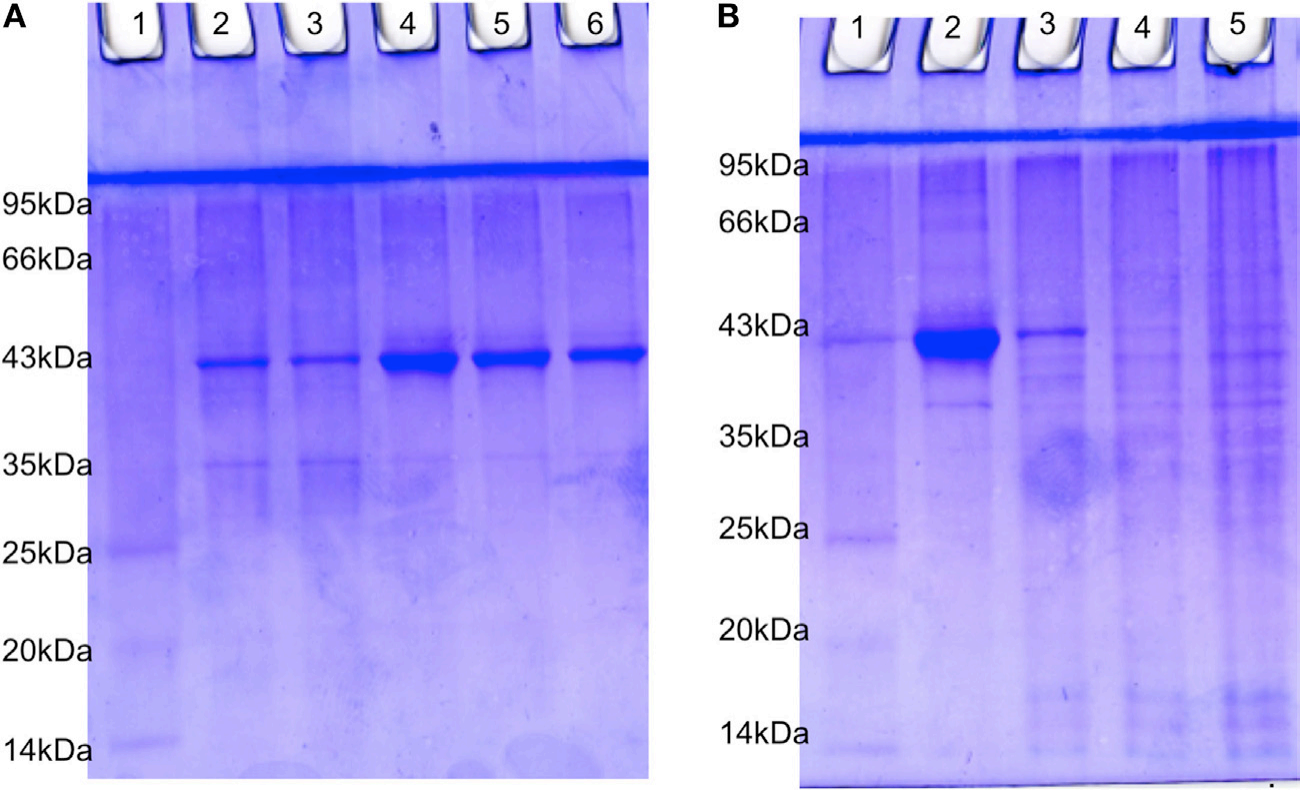
SDS-PAGE analysis of 15 µg protein extracted from **(A)** RS-307 strain of *Acinetobacter baumannii* cultured in Dulbecco’s Modified Eagle Medium (Lane 2, 3) and RS-307 cocultured and isolated from A-549 cell line, i.e., internalized bacteria (Lane 4–6). **(B)** A-549 cell line (Lane 2) as well as A-549 cell line infected with carbapenem-resistant strain RS-307 of *A. baumannii* (Lane 3–5). Lane 1 of both the gels represent protein marker.

The estimated value of protein isolated from suspension bacteria, internalized bacteria, cell line blank, and coinfected cell line were found to be 4.20, 1.51, 2.0, and 1.77 µg/µl, respectively. The SDS-PAGE profiling of RS-307 bacterial proteins (Figure 7A) and A-549 cell line proteins (Figure 7B) in the presence and absence of each other. The result confirms that after coinfection, protein with 43 kDa overexpressed while a protein with 35 kDa undergoes downregulation in the protein profiling of bacteria (Figure 7A). Similarly after infection a protein about 40 kDa expression is markedly decreased and banding pattern of A549 is totally different after *Acinetobacter* infection.

## Discussion

*Acinetobacter baumannii* is a Gram-negative multi drug-resistant bacterium causing nosocomial infection. Emergence of drug resistance in *A. baumannii* will lead to the high mortality and morbidity. Therefore, it is a high time to develop an alternative drug against carbapenem-resistant strain of *A. baumannii*. There are different approaches including herbal-based (5, 20, 21), nanomaterial-based (12, 22) and combination therapy (23–29), which have been tried recently against *A. baumannii* and few of them have shown very promising results.

Silver nanoparticles have recently emerged as antimicrobial agents for treating bacterial infections. PVP-capped AgNPs (11, 12) and citrate-capped AgNPs (22) were found to inhibit the growth of *A. baumannii*. Interaction of AgNPs with different cell models and their cellular effect have been reviewed recently (30) and showed the involvement of ROS during interaction of AgNPs with different cell lines. Recently, it is also reported that acinetin-505, a small lipopeptide-like compound, have role in the interaction of *A. baumannii* with pulmonary cells model (31). Most of the studied done so far have highlighted the use of AgNPs against *A. baumannii* in suspension form but less have been studied about the inhibitory role of AgNPs in the infection of *A. baumannii* to the human cell model.

Therefore, we have chemically synthesized PVP-capped AgNPs and characterized them for size and zeta-potential. The results confirm the formation of PVP-capped AgNPs with size around 100 nm. Synthesized nanoparticles were found active against carabpenem-resistant strain of *A. baumannii*, hence further used for its effect during infection of *A. baumannii* to human pulmonary cells model. Result of coinfection showed that *A. baumannii* leads to the death of basal epithelial cell line, i.e., A-549 and infection of *A. baumannii* to human pulmonary cells was inhibited by PVP-AgNPs. Similarly, PVP-AgNPs have no or very less cytotoxic effect at the bacterial inhibitory concentration. Likewise, it was also seen that there is a decrease of 80% viability of the intracellular bacteria. Therefore, present study suggest that PVP-AgNPs can be developed as a good alternative to carbapenem (beta-lactam), which inhibit the growth of carbapenem-resistant strain of *A. baumannii*.

## Conclusion

*Acinetobacter baumannii* causes pneumonia *via* targeting human pulmonary cells. Hence, human alveolar basal epithelial cell line A-549 was selected as a model to study the infection caused by *A. baumannii*. The present study concludes that during infection of *A. baumannii* about 40% bacteria adhere to the surface of A549 while about 20% get internalized inside the pulmonary cell line. We have also seen that during *Acinetobacter* infection, ROS concentration was found to increase by threefold. We have synthesized PVP-capped AgNPs using chemical methods and tested its efficacy against carbapenem-resistant strain of *A. baumannii*. The result showed that 30 µM PVP-AgNPs inhibit growth of *A. baumannii* in *in vitro* experiments. We have also shown that this concentration of PVP-AgNPs does not show any cytotoxic effect on the human pulmonary cell line with IC_50_ value of 130 µM. The PVP-AgNPs treatment causes about 80% decrease in the viability of the intracellular bacteria. Therefore, based on the result of the present study it can be concluded that PVP-capped silver nanoparticle can be a suitable replacement to the current antibiotics used against *A. baumannii*. Pulmonary cell targeted delivery of PVP-AgNPs in animal model can be further studied to use this molecule as a suitable drug against *A. baumannii*.

## Author Contributions

Conceived and designed the experiments, analyzed the data, wrote the manuscript: VT, Performed the experiments: VT, MT, and VS. Proofread of final version: VT, and MT.

## Conflict of Interest Statement

The authors declare that the research was conducted in the absence of any commercial or financial relationships that could be construed as a potential conflict of interest.
